# No Reduction in Parastomal Hernia Rate 3 Years After Stoma Construction With Prophylactic Mesh: Three-year Follow-up Results From STOMAMESH—A Multicenter Double-blind Randomized Controlled Trial

**DOI:** 10.1097/SLA.0000000000005537

**Published:** 2022-07-15

**Authors:** Christian Ringblom, Christoffer Odensten, Karin Strigård, Ulf Gunnarsson, Pia Näsvall

**Affiliations:** * Department of Surgical and Perioperative Sciences, Umeå University, Umeå, Sweden; † Sunderby Research Unit, Umeå University, Luleå, Sweden

**Keywords:** parastomal hernia, mesh, prevention, prophylaxis

## Abstract

The primary objective was to compare rates of parastomal hernia (PSH) 3 years after stoma construction with prophylactic mesh or no mesh. A secondary objective was to compare complications requiring reintervention within 3 years.

**Background:**

Recent studies have shown that a prophylactic mesh does not reduce the rate of PSH contrary to older studies. Long-term data on efficacy and safety is however scarce.

**Methods:**

A randomized controlled double-blind multicenter trial. Patients planned for permanent end colostomy were randomized to either prophylactic mesh in the retromuscular position around the stoma site or no mesh. They were evaluated for PSH clinically and with computed tomography (CT) 3 years after stoma construction. Medical records of all patients included were also reviewed at 3 years to detect any abdominal or abdominal wall surgery during that period.

**Results:**

A total of 232 patients were randomized. At 3 years, 154 patients were available for clinical evaluation and 137 underwent a CT scan. No significant difference in PSH rates was seen between the treatment allocation arms (clinical: *P*=0.829 and CT: *P*=0.761, respectively), nor was there a significant difference in the number of reinterventions, but 2 patients had their mesh removed at emergency surgery.

**Conclusions:**

Prophylactic mesh does not reduce the rate of PSH and cannot be recommended for routine use.

Parastomal hernia (PSH) is a common long-term complication after stoma construction and studies have reported PSH rates up to 50%.^1^,^2^ Results vary due to differences in follow-up time, definition of PSH, method used for detection of PSH, and type of stoma.

Although previous studies have shown a negative impact on quality of life (QoL) and body image experience among patients with a PSH^3^,^4^ many patients do not undergo surgical repair which could be explained by a recurrence rate of 6% to 34%^5^,^6^ and mortality rate of 0% to 6%.^5^,^7^ For this reason, research on the prevention of PSH is highly relevant.

One method said to reduce PSH is the placement of a prophylactic mesh at the time of stoma construction. Several randomized controlled trials (RCTs) have presented results that support the use of this approach to reduce the rate of PSH.^8^–^14^ In 2017, the European Hernia Society strongly recommended the use of prophylactic mesh when constructing a colostomy.^15^ However, 3 recent large RCTs could not confirm the advantage of mesh over no mesh at 1- or 2-year follow-up.^16^–^18^ Complication rates were similar in both groups. Only 2 RCTs had a median follow-up >3 years.^9^,^12^

In 2016, Kokotovic et al^19^ published long-term results following incisional hernia repair in Denmark showing that the risk for mesh-related complications requiring surgical intervention continue to increase well after 1 year. This further emphasizes the importance of long-term evaluation of safety aspects when using prosthetic mesh for PSH prevention.

The STOMAMESH trial was initiated to shed light on the outcome of using prophylactic mesh in stoma construction to prevent PSH. STOMAMESH is a double-blind multicenter RCT comparing mesh with no mesh and is currently, to our knowledge, the largest RCT investigating the efficacy of prophylactic mesh in preventing PSH. Odensten et al^16^ published 1-year follow-up results from this trial and concluded, contrary to previous studies, that there was no risk reduction for PSH when using a mesh. No significant difference in early complication rates was found between the 2 groups. In 2019, Näverlo et al^20^ reported the QoL of patients in the STOMAMESH trial after 1 year and found no difference in overall QoL between groups.

The present study provides outcome results of the STOMAMESH trial at 3 years. The primary outcome variable was the rate of PSH, and a secondary outcome was the rate of complications requiring surgical intervention.

## METHODS

### Study Design and Patients

STOMAMESH was designed as a double-blind multicenter RCT where patients are randomized to either prophylactic mesh or no mesh when receiving a permanent colostomy. Eight Swedish hospitals participated in the study. Inclusion criteria were the age of 18 years and older, no previous stoma, and signed informed consent. Exclusion criteria were fecal peritonitis, expected survival of <3 years or no informed consent. The study adhered to the CONSORT 2010 criteria for RCTs.

### Randomization

Randomization was carried out using opaque, sealed envelopes stratified to each hospital in blocks of 4. This procedure was carried out externally by the Northern Regional Cancer Center, Umeå University Hospital, and was blinded to the participating hospitals. Each participating hospital received 100 envelopes. In the case of protocol violation or treatment other than that specified in the envelope, a further 3 new patients were included to maintain power.

### Sample Size

At the time of study design, an incidence of PSH with a mesh of 5% versus 20% without was assumed. It was also expected that the majority of included patients would be operated for a colorectal malignancy with an expected 5-year survival just exceeding 50%. To achieve a power of 80% at a 95% significance level, the study would need to include a total of 220 patients.

### Surgical Procedures

#### Nonmesh Group

The stoma site was marked out before surgery. The bowel was taken through the rectus muscle and fixated to the skin with interrupted monofilament mucocutaneous sutures.

#### Mesh Group

The surgical technique previously described by Jänes et al^21^ was used and an instruction video was sent to the participating hospitals. The stoma site was marked out before surgery. A 10 × 10 cm lightweight (25–40 g/m^2^) polypropylene mesh was placed in the space posterior to the rectus muscle and anterior to the posterior rectus sheath. The mesh was anchored laterally with single nonabsorbable monofilament sutures and medially with the continuous suture used for closing the midline. A cruciate incision in the center of the mesh was made and the bowel taken out through the mesh and rectus muscle. The stoma was then fixated to the skin with interrupted monofilament mucocutaneous sutures.

### Endpoints and Data Collection

Patients were followed up for PSH at 3 years using computed tomography (CT) scanning and clinical evaluation. All reports were made prospectively on individual case report forms. Early complications and follow-up at 1 year have been reported previously (Odensten and colleagues, 2017). To ensure complete reporting of complications, the medical records of each patient included were examined at each participating hospital by the first author (C.R.) for the period up to 3 years after index surgery. Any surgical intervention (Clavien-Dindo grade 3b)^22^ involving the abdominal cavity or abdominal wall within 3 years after stoma construction was noted.

Patients requiring surgery for PSH were recorded as having a PSH and not followed up further. Patients undergoing surgery where the stoma was moved for reasons other than PSH were not included in further clinical or radiological follow-ups.

### CT and Clinical Follow-up

CT scans of the abdomen were carried out in the supine position without the Valsalva maneuver. Scans were evaluated by a radiologist and classified according to the Moreno-Matias scale.^23^

Clinical examinations were performed by a surgeon with the patient in the supine and standing position, with and without the Valsalva maneuver. The examiner reported if a PSH was present. In cases where a bulge was found around the stoma but not judged as a clinical hernia, the examiner had the possibility to report this as a “bulge only.” This parameter was defined as a protrusion around the stoma seen by the surgeon or patient but not deemed to be a hernia on clinical examination. A “bulge only” was not coded as a hernia and for the purposes of this study, only reported hernias were used for analysis. Both the patient and the examiner were blinded to treatment allocation, that is, whether a prophylactic mesh had been implanted or not.

### Statistical Analysis

Statistical analyses were carried out using RStudio, v1.3.1093 including the tidyverse package. The χ^2^ test was used for categorical variables and the Student independent *t* test for continuous variables. A *P* value <0.05 was considered statistically significant.

### Ethics Approval

The study protocol adhered to the Helsinki Declaration and was approved by the Regional Ethics Committee at Umeå University, Sweden (DNR 07-081 M). The study was registered at ClinicalTrials.gov (Identifier: NCT00917995).

## RESULTS

**FIGURE 1:**
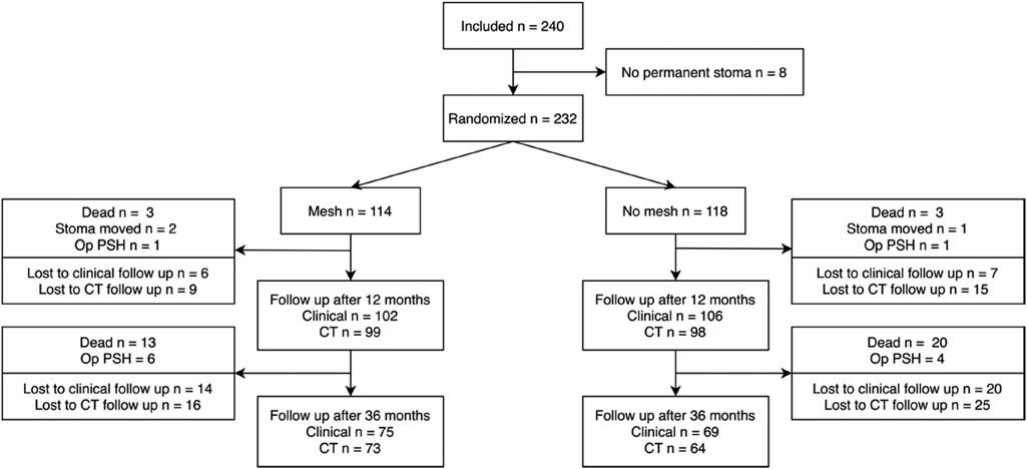
Flowchart.

A total of 240 patients were included between December 2007 and October 2015 (Fig. 1). Eight of these were excluded as they did not receive a permanent stoma. The remaining 232 patients were randomized to either mesh (n=114) or no mesh (n=118). Two patients randomized to the mesh group did not receive a mesh due to technical difficulties during the procedure. One patient in the no mesh group received a mesh for reasons unknown. These 3 patients were followed up in their previously allocated group according to protocol. At the 3-year follow-up, 154 patients (66.4%) were available for analysis (81 in the mesh group and 73 in the no mesh group) giving a de facto power of 80% at 3 years when using parameters from the original sample size calculation.^16^

At the 3-year follow-up, there was no significant difference in the rate of PSH clinically between the treatment groups (Table 1). Similarly, no significant difference was observed between the groups when examined by CT scan and classified according to the Moreno-Matias scale^23^ (Table 2).

**TABLE 1:** Rate of PSH at 3-year Clinical Follow-up

n (%)		
No Mesh (N=107)	Mesh (N=103)	*P*
40 (37.4)	40 (38.8)	0.829

Patients who underwent surgery for PSH were included as having a hernia.

**TABLE 2:** Rate of CT Confirmed PSH at 3-year Follow-up

	n (%)		
Highest Moreno-Matias Score	No Mesh (N=101)	Mesh (N=99)	*P*
2–3	34 (33.7)	32 (32.3)	0.840
1–3	45 (44.6)	42 (42.4)	0.761

There were no significant differences in baseline characteristics (Table 3) between the groups. After 3 years, 7 patients had undergone surgery for PSH in the mesh group and 5 in the no mesh group. These patients were recorded as having a hernia at clinical follow-up and not further evaluated with CT. A further 3 patients (2 in the mesh group and 1 in the no mesh group) were not followed up as they had had their stoma moved before the 1-year follow-up. The 2 patients in the mesh group had the mesh removed at stoma relocation.

**TABLE 3:** Baseline Characteristics of Study Population

	n (%)			
	No Mesh (N=118)	Mesh (N=114)	*P*	Missing
Sex, male	62 (52)	74 (65)	0.056	0
Age [mean (range)] (y)	69.8 (35–89)	69.7 (41–86)	0.906	0
ASA 1+2	79 (67)	82 (72)	0.510	8
ASA 3	34 (29)	29 (25)	0.510	
BMI [mean (range)]	26.3 (18.5–43.7)	26.1 (16.7–37.8)	0.725	6
Smoker	8 (7)	12 (11)	0.326	16
Malignancy	103 (87)	107 (94)	0.123	1
Elective surgery	118 (100)	113 (99)	0.308	0

ASA indicates American Society of Anesthesiologists.

Sixteen patients (14.0%) in the mesh group and 23 (19.5%) in the no mesh group died within 3 years after surgery. At the 1-year follow-up, although being eligible, 24 patients in the mesh group and 13 in the no mesh group did not undergo clinical and CT scan examinations. Corresponding figures at 36 months were 41 and 36 patients respectively. Reasons for loss to follow-up are summarized in Table 4.

**TABLE 4:** Reasons for Patients Being Lost to Follow-up Although Eligible

	1-year Clinical		3-year Clinical		1-year CT		3-year CT	
Reason	Mesh	No Mesh	Mesh	No Mesh	Mesh	No Mesh	Mesh	No Mesh
Did not want to continue	1	3	1	4	1	3	1	3
Palliative care or treatment	0	3	1	5	0	4	1	5
Did not turn up to follow-up	2	0	3	3	2	1	3	5
Dementia	1	0	1	0	1	0	1	0
Not reported	2	1	8	8	5	8	10	12

A total of 39 surgical procedures (Table 5) involving the abdominal cavity and abdominal wall on 29 patients were reported, including surgery for PSH (25 operations on 16 patients in the mesh group vs 14 operations on 13 patients in the no mesh group, *P*=0.15). Seven patients underwent surgery for PSH in the mesh group (1 patient had surgery twice, 1 patient had emergency surgery), and 5 in the no mesh group. Eight patients (4 in each group) had surgery due to a local stomal complication (stomal necrosis, bowel fistula, or abscess). Eight patients (4 in the mesh group, 3 in the no mesh group) required surgery for bowel obstruction, none due to PSH. Three patients in the mesh group were reoperated for bleeding, and 2 patients in the no mesh group were reoperated for midline wound infection and extraction of a suprapubic catheter.

**TABLE 5:** Frequency of Abdominal Cavity or Wall Surgery Within 3 Years in Each Group Categorized by Indication for Surgery

Reason for Surgery	No Mesh Group (N=118)	Mesh Group (N=114)	*P*
PSH	5	8	
Local stomal complication	4	8	
Bowel obstruction	3	6	
Hemorrhage	0	3	
Other	2	0	
Total	14	25	0.153

Values are presented as total surgical procedures per category. Header values (N) are number of patients per group.

## DISCUSSION

The conclusion of this large multicenter double-blind RCT is that the use of prophylactic mesh does not reduce the risk for PSH within 3 years after the construction of a stoma. No significant difference in the rate of abdominal or abdominal wall surgery was seen between the groups during this period. The present study confirms the 1-year outcome of the same trial presented by Odensten et al.^16^ As expected, the cumulated rate of PSH had further increased at the 3-year follow-up (n=79 clinical, n=77 CT) compared with the figures at 1 year (n=62 clinical, n=69 CT)^16^ showing that the number of PSH increases with time, though the majority of hernias occur within the first year after surgery. This is comparable with the GRECCAR7^18^ trial where 70% of the hernias were seen within the first year of the 2 years follow-up period. On the contrary, in the PREVENT trial, only 39% of hernias were seen within the first year. This can partly be explained by the longer follow-up period of 5 years but also due to the low PSH rate of 14% after 1 year compared with that of the other recent RCTs.^16^–^18^

Two previous RCTs with a similar or longer follow-up period^9^,^12^ did not evaluate the presence of PSH with CT. In the present study, the clinical PSH rate in the control group was lower than that reported by those 2 studies. This could be explained by the fact that, contrary to the other studies, the occurrence of bulging alone was not reported as a hernia in the present study. Comparison of studies evaluating PSH is generally problematic due to differences in definition and evaluation methods.

A strength of this study is the use of blinded examiners. Neither the Dutch PREVENT trial reported by Brandsma et al^10^ nor the Norwegian multicenter RCT reported by Lambrecht et al,^12^ both favoring the use of mesh, had blinded examiners. On the contrary, 2 recent RCTs,^17^,^18^ both with examiner blinding, showed no reduction in PSH when using a mesh. This would seem to suggest that clinical evaluation of PSH is prone to examiner bias.

In the present study, 79 patients were found to have a PSH. Despite this, only 12 (15.2%) underwent repair, probably because the symptoms of most PSHs do not motivate the risks of repair. The number of patients undergoing surgical repair of a PSH were similar between the 2 treatment arms (7 with mesh vs 5 with no mesh) which is in line with previous studies reporting this outcome^12^–^14^,^18^ suggesting that the dignity of symptoms from PSH are similar regardless of mesh or no mesh.

The numbers of patients who underwent a surgical procedure of the abdomen or abdominal wall were similar between the 2 groups (16 with mesh vs 13 with no mesh). Worthy of note is that when reviewing the medical records of the patients included, we discovered 2 patients in the mesh group that had had their mesh removed at emergency surgery during the first year, one for bowel fistulation and the other parastomal abscess. These were not reported and thus not included in the results presented by Odensten et al.^16^ These are rare complications and have not been described previously in similar studies. This may be explained by smaller population sizes and different local strategies for managing mesh-related infection. The population size in this study was not large enough to draw any conclusion on whether mesh increases the risk for local stomal complication requiring surgical intervention since this is a rare event. However, it is a concern that mesh removal was deemed necessary in 2 of our cases.

All procedures in this study were carried out using an open technique. This can be explained by 2 reasons: First, open surgery was the preferred technique for rectal cancer surgery in Sweden during the study period. Second, the described surgical technique for mesh implantation in this study required a midline incision. As stoma construction, with or without a sublay mesh, is independent of the surgical technique it would be reasonable to assume similar outcome for the rate of PSH when comparing open and laparoscopic procedures. In a recent RCT^17^ which included both laparoscopic and open procedures, no difference in PSH rate was seen between the open and laparoscopic approach.

In this study a keyhole technique was evaluated for PSH prevention, however, in the case of repair, a systematic review did show results favoring the Sugarbaker technique over a keyhole repair although no large prospective trials were found. It would certainly add to the field of PSH prevention to evaluate the efficacy of this technique. However, when taking into account concerns about the long-term risks involved with the large mass of intraperitoneal mesh used in the Sugarbaker approach, it would be reasonable to await long-term results on the safety of this technique before using it in the prophylactic context.

### Limitations

A weakness of this trial is that we only compared PSH rate between groups. PSH mostly poses a risk of being symptomatic and a highly relevant outcome measure is QoL which the present study did not investigate. QoL figures from the STOMAMESH trial at the 1-year follow-up was reported by Näverlo et al^20^ showing no significant difference in overall QoL between the groups. Results from the PREVENT trial^10^ where only the patients were blinded also showed no difference in QoL despite a difference in PSH rates.

Odensten et al^16^ compared the STOMAMESH population with a cohort of patients which met the inclusion criteria but were not included. A lower proportion of American Society of Anesthesiologists 3 patients were found in the STOMAMESH population even when excluding patients who underwent emergency surgery (28.1% vs 47.2%, *P*=0.002) implying that an unintended selection may have been present.

Previous studies have shown a positive relationship between body mass index (BMI) and PSH rate^2^,^24^ suggesting that there could be a theoretical advantage of using a prophylactic mesh in a cohort with a higher BMI. The current study, based on routine surgery, is underpowered to answer such a hypothesis for a specific subgroup with only 36 patients exceeding a BMI of 30 kg/m^2^.

## CONCLUSIONS

The use of prophylactic mesh in the retromuscular position when constructing a stoma does not reduce the risk for PSH. Together with the recently published RCTs by Prudhomme and colleagues and Marinez and colleagues that came to the same conclusion, should warrant the European Hernia Society to review their guidelines from 2017^15^ regarding the use of prophylactic mesh when constructing a stoma.
